# Doppler waveform study as indicator of change of portal pressure after administration of octreotide

**DOI:** 10.12669/pjms.324.10275

**Published:** 2016

**Authors:** Shahbaz Haider, Qurban Hussain, Sumera Tabassum, Bilal Hussain, Muhammad Rasheed Durrani, Fayyaz Ahmed

**Affiliations:** 1 Dr. Shahbaz Haider, FCPS. Department of Medicine, Jinnah Postgraduate Medical Centre (JPMC), Karachi Pakistan; 2 Dr. Qurban Hussain, FCPS. Department of Medicine, Jinnah Postgraduate Medical Centre (JPMC), Karachi Pakistan; 3 Dr. Sumera Tabassum, FCPS. Department of Radiology, Jinnah Postgraduate Medical Centre (JPMC), Karachi Pakistan; 4 Dr. Bilal Hussain, MBBS. Department of Medicine, Jinnah Postgraduate Medical Centre (JPMC), Karachi Pakistan; 5 Dr. Muhammad Rasheed Durrani, FCPS. Department of Medicine, Jinnah Postgraduate Medical Centre (JPMC), Karachi Pakistan; 6 Dr. Fayyaz Ahmed, MBBS. Department of Radiology, Jinnah Postgraduate Medical Centre (JPMC), Karachi Pakistan

**Keywords:** Doppler waveform, Octreotide, Hepatic vein, Portal hypertension

## Abstract

**Objective:**

To estimate the effect of portal pressure lowering drug ‘octreotide’, by observing the Doppler waveform before and after the administration of intravenous bolus of octreotide and thus to assess indirectly its efficacy to lower the portal pressure.

**Methods:**

This quassi experimental study was carried out in Medical Department in collaboration with Radiology Department of Jinnah Postgraduate Medical Center Karachi Pakistan from September 10, 2015 to February 5, 2016. Cases were selected from patients admitted in Medical Wards and those attending Medical OPD. Diagnosis of cirrhosis was confirmed by Clinical Examination and Lab & Imaging investigation in Medical Department. Doppler waveform study was done by experienced radiologist in Radiology Department before and after administration of octreotide. Doppler signals were obtained from the right hepatic vein. Waveform tracings were recorded for five seconds and categorized as ‘monophasic’, ‘biphasic’ and ‘triphasic’. Waveform changes from one waveform to other were noted and analyzed.

**Results:**

Significant change i.e. from ‘monophasic’ to ‘biphasic’ or ‘biphasic’ to ‘triphasic’ was seen in 56% cases while ‘monophasic’ to ‘triphasic’ was seen in 20% cases. No change was seen in 24% cases. Improvement in waveform reflects lowering of portal vein pressure.

**Conclusion:**

Non invasive Hepatic vein Doppler waveform study showed improvement in Doppler waveform after administration of octreotide in 76% cases. Doppler waveform study has the potential of becoming non invasive ‘follow up tool’ of choice for assessing portal pressure in patients having variceal bleed due to portal hypertension.

## INTRODUCTION

Hepatic Vein pressure Gradient (HVPG) is usual standard way of assessing portal vein pressure in patients having liver cirrhosis.^1^,^2^ As measuring HVPG is an invasive procedure, alternative methods, as observing hepatic vein Doppler waveform, are also being studies in cirrhotic and non cirrhotic patients.^3^,^4^ In normal persons hepatic vein Doppler waveform is ‘triphasic’, having two phases of higher and lower velocity and third phase of reversal of flow.^5^,^6^ In cirrhotic patients, waveform may be ‘biphasic’ with absence of normal reversal flow phase or may be ‘monophasic’ i.e. having only one phase in addition to normal ‘triphasic’ which may be seen in patients of cirrhosis during early stages.^7^

It has been shown by determining HVPG and observing waveform after administration of portal pressure lowering drugs like terlipressin, that from ‘monophasic’ to ‘biphasic’ or ‘biphasic’ to ‘triphasic’, HVPG change (decrease) was always 3 mm of Hg or more.^8^ Mean decrease in HVPG on improvement from ‘monophasic’ waveform to ‘biphasic’ waveform observed was 5.8 mm of Hg (range 3-10), while with two stepped wave change i.e. from ‘monophasic’ to ‘triphasic’ change in HVPG was seven (range 5-9). Some other studies have observed that Doppler waveform can be used to see the effect of portal vein pressure lowering drugs as an alternative method to invasive method of measuring HVPG.^5^,^8^

The objective of this study was to estimate the effect of portal pressure lowering drug ‘octreotide’, a somatostatin analogue, by observing the Doppler waveform before and after the administration of intravenous bolus of octreotide and thus to assess indirectly its efficacy to lower the portal pressure.

## METHODS

The study protocol was approved by the ethical committee of JPMC. All patients gave written informed consent to participate after complete explanation of the purpose of the study. This quassi experimental study was carried out in Medical Department in collaboration with Radiology Department of Jinnah Postgraduate Medical Center Karachi Pakistan from September 10, 2015 to February 5, 2016. Study was continued till 50 cases were enrolled and studied.

Cases were selected from patients admitted in Medical Wards and those attending Medical OPD. Informed written consent was taken from all ‘willing to be enrolled’ in study. Doppler waveform study was performed in Radiology Department. Patients of liver cirrhosis having either ‘monophasic’ or ‘biphasic’ waveform of hepatic vein on Doppler ultrasound were selected. Patients with ‘triphasic’ waveform on Doppler ultrasound were excluded as no improvement was expected to be recordable after administration of ‘octreotide’.

Diagnosis of cirrhosis was confirmed by Clinical Examination, Lab data, imaging studies using ultrasound and CT scan, ascitic fluid examination, SAAG value, upper Gastrointestinal endoscopy and histopatholgy. Patients who refused consent or had hepatocellular carcinoma or hepatic encephalopathy, high serum bilirubin (>5 mg \100 ml), thrombus in portal vein or in hepatic vein or inferior vena cava, constrictive pericarditis or congestive cardiac failure or any other disease causing dilated inferior vena cava were excluded. Patients haemodynamically unstable were also excluded. It was assured that patient is not taking any drug affecting portal pressure for preceding ten days.

After selection, and fulfilling the requirements, Doppler waveform study was done by experienced radiologist. Ultrasound machine Volusion 730 Pro V Kretz Austria was used. 35 M Hertz Convex probe was placed intercostally. Doppler signals were obtained from the right hepatic vein at a distance of four cm away from the junction of hepatic vein and inferior vena cava.

Hepatic vein Doppler waveform were observed for 20 minutes before injection ‘octreotide’ and tracing with best waveform was recorded and saved. Then injection ‘octreotide’ 50 microgram intravenous stat was given through a 20 gauge cannula on upper extremity and waveform was observed again for 20 minutes and best waveform was recorded and saved. All the tracings recorded were of 5-6 seconds duration with breath held at end expiration. Tracings were categorized by two radiologists having three years of experience in the field.

Waveform recordings were categorized as:

’monophasic’; flat line or wavy but no phasic change in amplitude of waves.

’biphasic’; phasic change in amplitude of waves seen but no reversal of flow (no third phase)

’triphasic’; phasic oscillation like that in ‘biphasic’ with addition of flow reversal phase.

Study was continued till Doppler waveforms of 50 patients were observed and recorded before and after administration of ‘octreotide’. These three categories were quantified by assigning 1 to ‘monophasic’, three to ‘biphasic’ and five to ‘triphasic’ waveform. By quantifying waveforms,’ change’ was also quantified by subtracting number of initial waveform from the number of later waveform. This change was defined as ‘significant’ if result of subtraction was ‘two’ and ‘gross’ if result of subtraction was ‘four’ i.e. if change was from ‘monophasic’ to ‘triphasic’ waveform. Statistical analysis was performed on SPSS 17. ‘p’ value was determined by applying wilcoxan signed ranksome matched pair test.

## RESULTS

Among the 50 patients who entered the study, 34 (68%) were male and 16 (32%) were female. Maximum number (n=18; 36%) belonged to age group 40-49 years followed by 10 (n=10;20%) in age groups of 30-39 and 60-69 years each. The mean age was 45.76 years. Results are tabulated in Table-I.

**Table-I:** Change in different types of waveform after administration of Injection Octrotide.

Initial Waveform N(%of total)	‘Monophasic’ N (% of initial waveform)	‘Biphasic’ N(%of initial waveform)	‘Triphasic’ N(%of initial waveform)
Monophasic’ 42(84%)	10(23.8%)	22(52.38%)	10(23.8%)
Biphasic 8(16%)	–––	2(25% of initial 8)	6(75% of 8)
Total 50(100%)	10(20%)	24(48%)	16(32%)

Significant change i.e. from ‘monophasic’ to ‘biphasic’ or ‘biphasic’ to ‘triphasic’ was seen in 28 (56%) cases while gross change i.e. from ‘monophasic’ to ‘triphasic’ was observed in 10 (20%) cases. So in 38 (76%) cases positive change either ‘significant’ or ‘gross’ was observed. No change was observed in 12 (24%) cases. Maximum detectable change (i.e. end point triphasic as better than it could not be observed by Doppler waveform) was seen in 16 (32%) cases. Wilcoxon signed Ranksome matched pair test was applied. Positive ranks were 38, ties were 12 and p value was < 0.005.

## DISCUSSION

Significant change i.e. ‘monophasic’ to ‘biphasic’ or ‘biphasic’ to ‘triphasic’ was seen in 28 (56%) cases after administration of octreotide. Acute variceal bleed is a life threatening medical emergency.^9^ To decrease the portal venous pressure by vasoactive drugs to stop variceal bleed is one of the important aims in management in addition to administration of blood products.^10^

As mentioned earlier, Doppler waveform of hepatic vein was used in the study to assess decrease in portal vein pressure. It is non invasive economical alternative of invasive method of HVPG, which is a standard although in itself it is also indirect method of estimating portal vein pressure as in this method measuring catheter does not enter portal vein. HVPG, is calculated after introducing venous catheter, reaching Hepatic vein and measuring its pressure, termed free pressure, when catheter is free floating in hepatic vein.^9^,^11^ Later catheter is moved forward, till it wedges in smaller hepatic vein. Pressure is again measured when catheter is in wedged position. HVPG is calculated after subtracting pressure ‘when catheter is free floating position’ from the pressure ‘when catheter is in wedged position’. But as HVPG measurement is invasive and costly, it is not always feasible and available for use, especially in developing countries like Pakistan.

Studies on hepatic vein Doppler waveform after administration of ‘octreotide’ are very scarce. We compared the results of this study with study by Baik^8^ done on same lines but the drug used in the study was ‘terlipressin’. Results of ours study using ‘octreotide’ and Baik’s study using ‘terlipressin’ are comparable and statistically not significantly different. Comparison of two drugs is shown in Table-II. As mentioned, studies on hepatic vein Doppler waveform after administration of Octreotide are hardly available, however studies done by measuring the HVPG after administration of Octreotide have shown that Octreotide reduces the HVPG.^11^ In study by Laurent,^11^ long acting octreotide showed persistent decrease in HVPG even after months. Studies by Baik and Jeong^12^ and Escorsell,^13^ showed that there was transient decrease in HVPG after administration of octreotide. A study by Moller^14^ showed no effect of octreotide on HVPG and portal flow. Study by Oberti^15^ showed that decrease in portal pressure was immediate and lasted for 60 minutes. Another Study^16^ showed octreotide reduces the azygos blood flow and it inhibits the postprandial increase in portal pressure in cirrhotic patients with portal hypertension.

**Table-II:** Comparison with study done by Baik SK et al.^8^

	Our study (octreotide) N (%)	Literature (terlipressin) {Baik} N (%)
Total patients n(%)	50(100%)	21(100%)
Gross change	10(20%)	3(14%)
Significant Change	28(56%)	15(71%)
Total showing change	38(76%)	18(85%)
No change	12(24%)	3(14%)

These are diverse findings though octreotide is widely studied for its effects on variceal bleeding and has been found effective for control of bleeding in cirrhosis.^9^,^10^,^17^ It has been assumed that HVPG measurement may not be true indicator of portal pressure,^18^ as portal vein is not entered. Diversity of results necessitates more insight into the issue. Like study by Baik,^8^ our one previous study^19^ on Doppler waveform change was done using the vasoactive drug “terlipressin” on similar lines. Results of previous and this study using different drugs i.e. ‘terlipressin and octreotide’ were also statistically compared and seen to be not significantly different.

### Limitation of the study

It was planned to see the effects of single dose of octreotide so it is not possible to comment on persistent effect of continuous infusion of octreotide. To observe the persistency or otherwise, of change of Doppler waveform with continuous infusion of octreotide, study may be planned with monitoring of hepatic vein waveform in Medical ICU or Medical Ward as prolonged stay in Radiology Department is not feasible.

## CONCLUSION

Non invasive Hepatic vein Doppler waveform study showed improvement in Doppler waveform after administration of octreotide in 76% cases. Doppler waveform study has the potential of becoming non invasive ‘follow up tool’ of choice for assessing portal pressure in patients having variceal bleed due to portal hypertension.
